# USP13 interacts with cohesin and regulates its ubiquitination in human cells

**DOI:** 10.1074/jbc.RA120.015762

**Published:** 2020-12-20

**Authors:** Xiaoyuan He, Jung-Sik Kim, Laura A. Diaz-Martinez, Cecil Han, William S. Lane, Bogdan Budnik, Todd Waldman

**Affiliations:** 1 Departments of Oncology, Biochemistry & Molecular Biology, Lombardi Comprehensive Cancer Center, Georgetown University School of Medicine, Washington, District of Columbia, USA; 2 Department of Biology, Gonzaga University, Spokane, Washington, USA; 3 Mass Spectrometry and Proteomics Resource Laboratory, Harvard University, Cambridge, Massachusetts, USA

**Keywords:** deubiquitylation (deubiquitination), chromatin, cell cycle, mitosis, protein–protein interaction, cell division, genome structure, cohesin, USP13, ac-SMC3, acetylated-SMC3, FSBP, FLAG-streptavidin-binding peptide, SBP, streptavidin binding peptide, TBS, Tris-buffered saline, USP13, Ubiquitin-Specific Protease 13, ZnF, zinc finger domain

## Abstract

Cohesin is a multiprotein ring complex that regulates 3D genome organization, sister chromatid cohesion, gene expression, and DNA repair. Cohesin is known to be ubiquitinated, although the mechanism, regulation, and effects of cohesin ubiquitination remain poorly defined. We previously used gene editing to introduce a dual epitope tag into the endogenous allele of each of 11 known components of cohesin in human HCT116 cells. Here we report that mass spectrometry analysis of dual-affinity purifications identified the USP13 deubiquitinase as a novel cohesin-interacting protein. Subsequent immunoprecipitation/Western blots confirmed the endogenous interaction in HCT116, 293T, HeLa, and RPE-hTERT cells; demonstrated that the interaction occurs specifically in the soluble nuclear fraction (not in the chromatin); requires the ubiquitin-binding domains (UBA1/2) of USP13; and occurs preferentially during DNA replication. Reciprocal dual-affinity purification of endogenous USP13 followed by mass spectrometry demonstrated that cohesin is its primary interactor in the nucleus. Ectopic expression and CRISPR knockout of USP13 showed that USP13 is paradoxically required for both deubiquitination and ubiquitination of cohesin subunits in human cells. USP13 was dispensable for sister chromatid cohesion in HCT116 and HeLa cells, whereas it was required for the dissociation of cohesin from chromatin as cells transit through mitosis. Together these results identify USP13 as a new cohesin-interacting protein that regulates the ubiquitination of cohesin and its cell cycle regulated interaction with chromatin.

Cohesin is a ubiquitously expressed protein complex that regulates 3D genome organization, sister chromatid cohesion, DNA replication, and DNA repair (1, 2, 3, 4, 5). Cohesin is a ring structure comprising four core subunits—SMC1, SMC3, RAD21, and either STAG1 or STAG2. Several additional subunits, PDS5A/B, WAPL, Sororin, NIPBL, and MAU2, regulate the core cohesin ring. Inherited mutations of cohesin subunits cause pediatric neurodevelopmental disorders known as cohesinopathies (6). Somatic inactivating mutations of cohesin subunits are common in diverse human cancer types (7, 8).

The cohesin complex is regulated by posttranslational modification. Acetylation of the SMC3 subunit is its most well-studied modification and is required for establishment of sister chromatid cohesion (9, 10). Cohesin subunits are also known to be ubiquitinated. In particular, Frattini *et al.* (11) have shown that cohesin subunits in *Saccharomyces cerevisiae* are ubiquitinated and this ubiquitination controls replication fork integrity and the cellular response to replication stress. Yeh *et al.* (12) have demonstrated that the WAPL subunit of human cohesin is ubiquitinated and that this can be controlled by the USP37 deubiquitinase. However, other than these findings, the mechanisms and effects of cohesin ubiquitination remain largely undefined.

Here we report that the USP13 deubiquitinase interacts with the human cohesin complex. We further define the mono- and poly-ubiquitination states of human cohesin subunits and demonstrate that USP13 is paradoxically (given its status as a *de*ubiquitinase) required for their ubiquitination. Finally, USP13-dependent ubiquitination of cohesin is required for the dissociation of cohesin from chromatin during mitosis.

## Results

### Interaction of endogenous USP13 with endogenous cohesin in cultured human cells

We recently used gene editing to introduce a FLAG/streptavidin binding peptide (SBP) dual epitope tag into either the amino or carboxyl terminus of each of 11 known components of cohesin in human cultured HCT116 cells. We then performed dual-affinity purification of nuclear extracts followed by mass spectrometry to identify novel cohesin-interacting proteins (13). In our initial report, we focused on splicing factors and RNA-binding proteins as novel cohesin-interacting proteins. However, those mass spectrometry data also identified Ubiquitin-Specific Protease 13 (USP13) as a putative novel cohesin-interacting protein.

**Figure 1:**
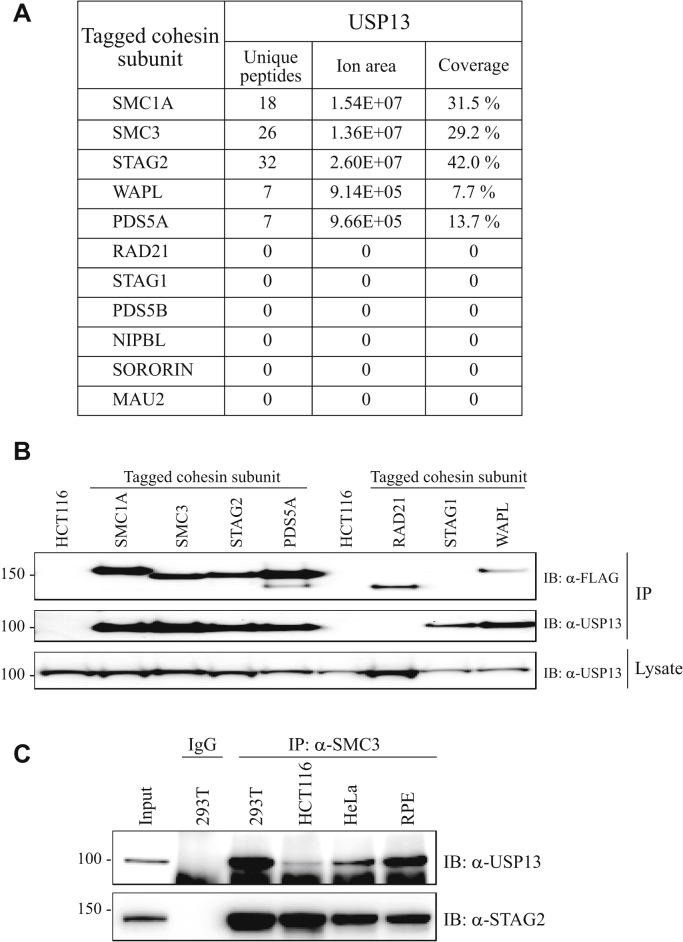
**Interaction of endogenous cohesin with endogenous USP13 in human cells.***A*, mass spectrometry was performed on dual-affinity purifications from gene-edited derivatives of HCT116 cells in which each of 11 cohesin genes was modified with the addition of a dual epitope tag. The number of unique peptides, ion area, and percent coverage of USP13 in each of the affinity purifications is shown. Ion area is considered a qualitative measure of relative protein abundance. All primary mass spectrometry data are in ref. (13). *B*, dual-affinity purifications from nuclear extracts of HCT116 parental cells and epitope-tagged derivatives were separated by SDS-PAGE and Western blot performed with antibodies to USP13 and FLAG (for detecting the bait protein in the affinity purifications). SMC1A, SMC3, STAG2, and PDS5A from 1 mg nuclear lysate; RAD21, STAG1, and WAPL from 2 mg nuclear lysate. *C*, immunoprecipitations with SMC3 antibodies (or IgG control antibodies) from 293T, HCT116, HeLa, and RPE-hTERT whole cell lysates were separated by SDS-PAGE and Western blot performed with antibodies to USP13 and STAG2.

USP13 was identified by mass spectrometry in 5 of 11 independent dual-affinity purifications, using dual epitope–tagged endogenous SMC1A, SMC3, STAG2, WAPL, and PDS5A proteins as baits (Fig. 1*A*). These mass spectrometry results were then confirmed by performing Western blot with USP13 antibodies on the same dual-affinity purifications (Fig. 1*B*). USP13 was also detected by Western blot in dual-affinity purifications from STAG1 epitope–tagged cells (Fig. 1*B*), despite not having been detected by mass spectrometry. Of note, USP13 is known to be present in both the nucleoplasm and cytoplasm of cultured human cells (14).

To further confirm the interaction between USP13 and cohesin, endogenous cohesin was immunoprecipitated with SMC3 antibodies from whole cell lysates prepared from genetically unmodified HCT116 cells and Western blot performed with USP13 antibodies (and STAG2 antibodies as a positive control). As expected, STAG2 was robustly coimmunoprecipitated by SMC3 (Fig. 1*C*). Endogenous USP13 was also robustly coimmunoprecipitated by SMC3, confirming the interaction between USP13 and cohesin in genetically unmodified cells. To demonstrate the generality of this interaction, similar immunoprecipitation/Western blots were performed on protein lysates from 293T cells, HeLa cells, and untransformed epithelial cells (RPE-hTERT). In each case, USP13 was robustly coimmunoprecipitated with endogenous cohesin complexes (Fig. 1*C*).

### Cohesin is the primary nuclear USP13-binding protein

At this point we had demonstrated that endogenous USP13 bound to endogenous cohesin in the nucleus of human cells. However, it was unclear whether cohesin was the *primary* USP13-binding protein in the nucleus (more interesting) or whether cohesin was *one of many* proteins that bound to USP13 in the nucleus. This question was particularly relevant since other groups have reported other proteins that could interact with USP13, including VPS34, IL-1R8, PTEN, MCL1, and GP78 (15, 16, 17, 18, 19). Therefore, we sought to determine the relative prominence of cohesin as a nuclear USP13-binding protein.

**Figure 2:**
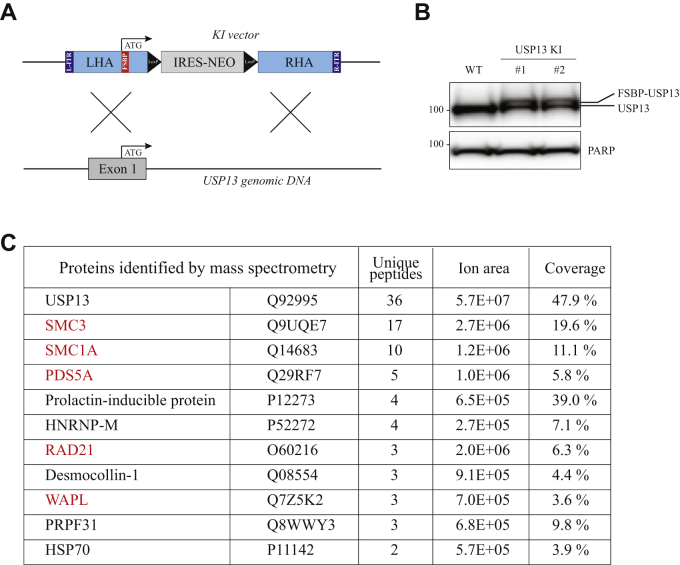
**Cohesin is the primary USP13 interactor in the nucleus.***A*, the USP13 epitope tagging vector was designed to modify the first coding exon (exon 1), adding a 1xFLAG-SBP (F-SBP) dual epitope tag immediately after the initiating methionine. Subsequent Cre-mediated recombination removed the FLOXed IRES-Neo^R^ gene in intron 1. *B*, Western blot with USP13 antibodies was performed on extracts from parental HCT116 cells and two independently derived heterozygous USP13 epitope-tagged derivatives. This confirmed the presence of the expected equimolar doublet, comprising untagged USP13 protein from the untargeted allele, and a slightly larger epitope-tagged USP13 protein from the epitope-tagged, targeted allele. *C*, mass spectrometry was performed on dual-affinity purifications from USP13 epitope-tagged cells (and parental control cells). All proteins present in affinity purifications from epitope-tagged cells but absent in affinity purifications from untagged parental cells are shown. The Uniprot accession number, number of unique peptides, ion area, and percent coverage are shown. Ion area is considered a qualitative measure of relative protein abundance. Known subunits of the cohesin complex are in *red*.

To identify nuclear USP13-associated proteins in an unbiased way, we used gene editing to introduce a FLAG-streptavidin-binding peptide (FSBP) dual epitope tag into the amino terminus of the gene encoding USP13 in HCT116 cells (Fig. 2*A*; see Experimental Procedures for details). The expression of a slightly larger epitope-tagged species of USP13 protein was confirmed by Western blot with USP13 antibodies (Fig. 2*B*). Nuclear extracts were then prepared from HCT116 cells and HCT116-FSBP-USP13 isogenic derivatives. Dual-affinity purification was then performed and analyzed by GeLC-MS/MS mass spectrometry (20).

As shown in Figure 2*C*, five of the top eight most abundant nuclear USP13-interacting proteins were components of cohesin—SMC1A, SMC3, WAPL, PDS5A, and RAD21. Of the other USP13-interacting proteins, two were splicing factors that we previously reported to be novel cohesin-interacting proteins (HNRNPM, PRPF31; ref. (13)). Of note, none of the proteins previously reported to interact with USP13 were confirmed as nuclear USP13 interactors in this analysis. Together, these data indicate that cohesin subunits are the primary nuclear USP13-interacting proteins.

### USP13 interacts with soluble cohesin complexes preferentially during DNA replication

**Figure 3:**
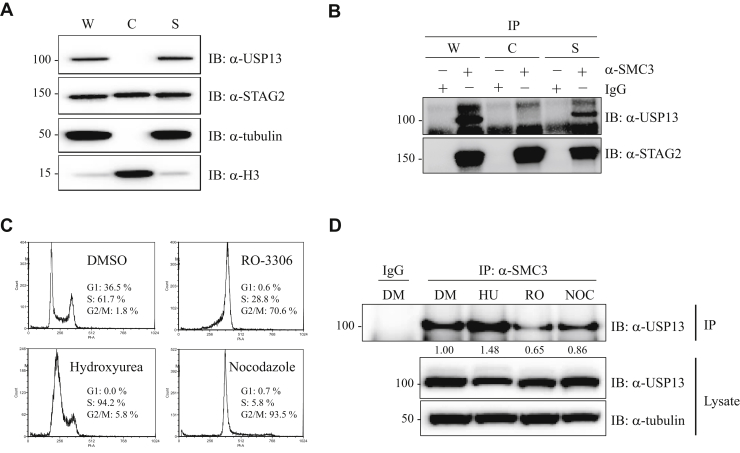
**USP13 interacts with soluble cohesin complexes preferentially during DNA replication.***A*, whole cell lysates (W), chromatin lysates (C), and soluble lysates (S) were prepared from 293T cells and Western blot was performed with antibodies to USP13, STAG2, tubulin (soluble protein), and histone H3 (chromatin protein). *B*, cohesin complexes were immunoprecipitated with SMC3 antibodies from the protein lysates described in *A*, and Western blot was performed with the antibodies indicated. *C*–*D*, 293T cells were treated for 20 h with dimethyl sulfoxide (DMSO) vehicle alone (DM); hydroxyurea (HU) to arrest cells in S phase; RO-3306 (RO) to arrest cells in G2; and nocodazole (NOC) to arrest cells in mitosis. *C*, flow cytometry was performed to document the cell cycle arrests. *D*, SMC3 immunoprecipitations were then performed on whole cell lysates, followed by Western blot with USP13 antibodies. Quantification of USP13 bands in SMC3 immunoprecipitates using ImageJ is shown.

In the nucleus, the cohesin complex exists in both chromatin-bound and chromatin-unbound (*i.e.*, soluble) states (13, 21). To determine whether USP13 interacts with chromatin-bound or -unbound cohesin complexes, 293T cell lysates were fractionated into chromatin-bound proteins and soluble proteins and Western blot performed with USP13 antibodies (Fig. 3*A*). As expected, cohesin (STAG2 subunit) was detected in both the chromatin and soluble fractions. However, USP13 was detected only in the soluble fraction, indicating that it must interact with cohesin in the soluble nuclear fraction, not in the chromatin fraction. To prove this, cohesin complexes were immunoprecipitated using SMC3 antibodies from 293T whole cell lysates, chromatin fractions, and soluble fractions. Western blot was then performed with antibodies to the STAG2 component of cohesin and USP13. SMC3 coimmunoprecipitated STAG2 from both the soluble and chromatin fractions, whereas USP13 was only coimmunoprecipitated from the soluble fraction (Fig. 3*B*). This experiment confirmed that USP13 interacts specifically with non–chromatin-bound cohesin complexes.

We next sought to determine whether the interaction between cohesin and USP13 was cell cycle dependent. To test this, 293T cells were arrested at different points in the cell cycle and the interaction between cohesin and USP13 measured by performing USP13 Western blots on cohesin immunoprecipitates. In particular, 293T cells were treated for 20 h with hydroxyurea to arrest cells in the S phase, RO-3306 (a CDK inhibitor) to arrest cells in G2, and nocodazole to arrest cells in mitosis (flow cytometry in Fig. 3*C*). SMC3 immunoprecipitations were then performed on whole cell lysates, followed by USP13 Western blot. As shown in Figure 3*D*, the interaction between cohesin and USP13 was enhanced in cells arrested in the S phase when compared with asynchronous cycling cells, or cells arrested at other points in the cell cycle. These data indicate that the interaction of USP13 with cohesin is cell cycle dependent—it is enhanced during DNA replication

### The ubiquitin-binding domains of USP13 are required for its interaction with cohesin

**Figure 4:**
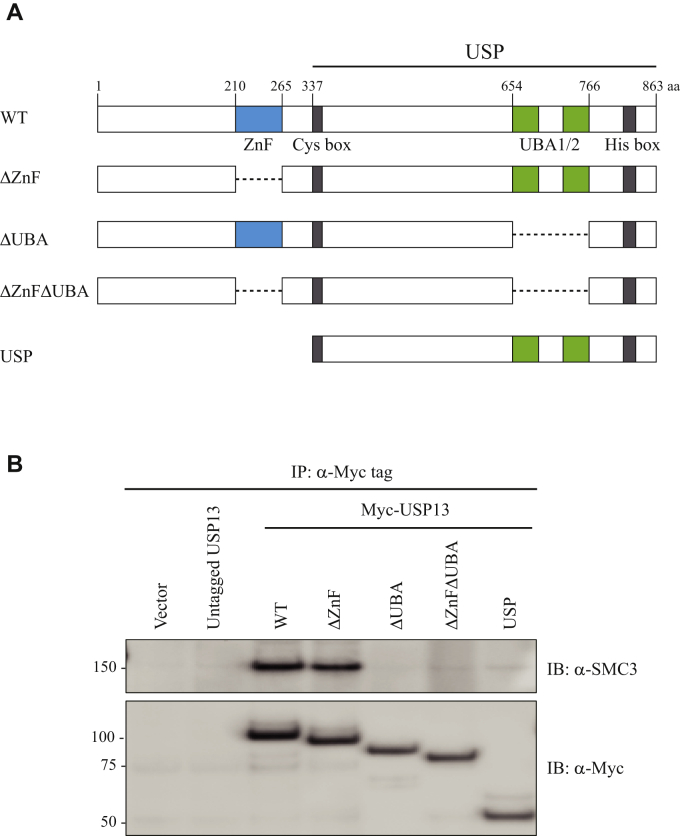
**Domain analysis of the interaction of USP13 with cohesin.***A*, human USP13 is an 863 amino acid protein with ZnF and UBA (ubiquitin-binding) functional domains, as well as with cysteine-rich and histidine-rich regions. The structure of derivatives lacking the ZnF and UBA1/2 functional domains individually and in combination are shown: ΔZnF (deletion of amino acids 210–265), ΔUBA (deletion of amino acids 654–766), and ΔZnFΔUBA. Also shown is a derivative comprising only the “USP” portion of the protein, thereby lacking virtually the entire amino terminal half of USP13. *B*, constructs were transfected into 293T cells and whole cell lysates prepared. Immunoprecipitations with myc antibodies were performed on equivalent amounts of protein (250 μ), followed by Western blot with myc antibodies (to confirm expression of the transfected constructs), and with SMC3 antibodies.

USP13 is a 97-kDa protein with two well-defined functional domains (Fig. 4*A*), a zinc finger domain (ZnF), and two adjacent ubiquitin binding domains (UBA1/2). We next sought to determine which (if either) of these domains was required for the interaction of USP13 with cohesin.

To do this, we modified a human USP13 expression vector (gift from Yihong Ye; ref. (19)) by adding a myc epitope tag to the amino terminus. We then transfected 293T cells and validated that immunoprecipitation of ectopically expressed USP13 protein with myc antibodies resulted in the coimmunoprecipitation of endogenous cohesin (Fig. 4*B*). We next generated three derivatives of the USP13 expression vector lacking the ZnF and UBA1/2 functional domains individually and in combination: ΔZnF (deletion of amino acids 210–265), ΔUBA (deletion of amino acids 654–766), and ΔZnFΔUBA (Fig. 4*A*). We also generated a derivative comprising only the “USP” portion of the protein, thereby lacking virtually the entire amino-terminal half of USP13. These constructs were transfected into 293T cells, whole cell lysates were prepared, immunoprecipitation was performed with Myc antibodies, and Western blot was performed with antibodies to the SMC3 subunit of cohesin (Fig. 4*B*). The USP13 protein lacking the UBA1/2 domains completely lost the ability to interact with cohesin, whereas the USP13 protein lacking its ZnF domain maintained its ability to interact with cohesin. The USP13 protein lacking the entire amino-terminal half of the protein similarly also lost its ability to interact with cohesin. These data demonstrate that the UBA1/2 domains are required for the interaction of USP13 with cohesin and that the ZnF domain is dispensable for the interaction of USP13 with cohesin.

### USP13 is dispensable for sister chromatid cohesion in human cells

The canonical function of cohesin is to cohere replicated chromosomes until it is cleaved to initiate the metaphase to anaphase transition. Inactivation of some, but not all, subunits of cohesin can result in abrogation of sister chromatid cohesion. Therefore, we tested whether USP13, as a new cohesin-interacting protein, was required for sister chromatid cohesion.

To test this, we used CRISPR-mediated gene editing to introduce biallelic inactivating mutations into USP13 in both HCT116 cells and HeLa cells (see Experimental Procedures for details), two human cancer cell lines with wildtype cohesin genes, and intact sister chromatid cohesion. Inactivation of USP13 in two independently derived KO clones for each cell line was validated by Western blot, as shown in Fig. S1*A*. Next, cells were treated with nocodazole to trap cells in mitosis, then stained with Giemsa. Sister chromatid cohesion was then assessed by microscopy, as described in detail in Experimental Procedures. As shown in Fig. S1*B*, mutational inactivation of USP13 had no effect on sister chromatid cohesion (or mitotic index) in either HCT116 cells or HeLa cells.

### Ubiquitination of endogenous cohesin subunits in cultured human cells

Since USP13 is an isopeptidase that regulates ubiquitin dynamics, we hypothesized that USP13 might regulate its newly discovered interacting cohesin subunits by deubiquitination. However, before it was possible to test this hypothesis, we first needed to define the ubiquitination state of endogenous cohesin subunits in cultured human cells.

**Figure 5:**
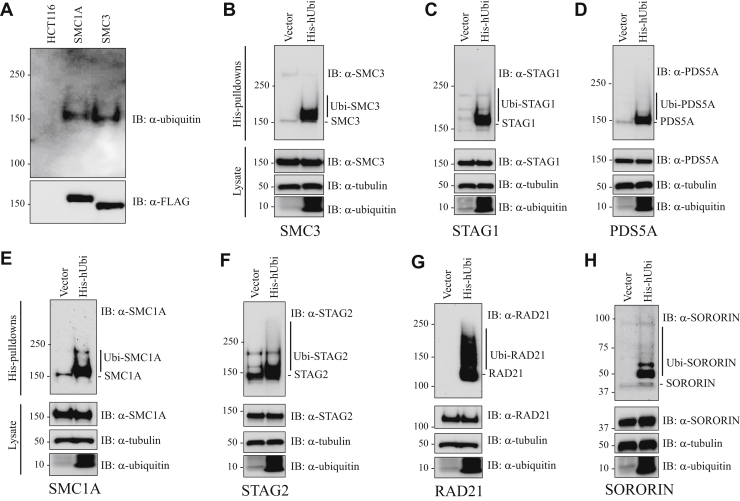
**Ubiquitination of cohesin in human cells.***A*, nuclear lysates (2 mg) from HCT116 parental cells and gene-edited derivatives in which a FLAG/SBP dual-epitope tag was engineered into the amino terminus of the endogenous SMC1A and SMC3 subunits of cohesin were purified by dual-affinity purification, and Western blot was performed with ubiquitin antibodies (Thermo Fisher). *B*–*H*, 293T cells were transfected with an expression vector for His-tagged ubiquitin (His-hUbi), or vector alone. Twenty-four hours after transfection, the MG132 proteasome inhibitor was added and the cells were cultured for an additional 4 h. The cells were then lysed in denaturing lysis buffer and sonicated and the ubiquitinated proteins purified by affinity purification on a Ni column, as described in detail in Experimental Procedures. The ubiquitinated proteins were then separated by SDS-PAGE, and Western blot was performed with antibodies to *B*, SMC3; *C*, STAG1; *D*, PDS5A; *E*, SMC1A; *F*, STAG2; *G*, RAD21; and *H*, Sororin.

Initially we felt that it was important to show that endogenous cohesin was ubiquitinated in untransfected human cells. To do this, nuclear lysates were prepared from HCT116 parental cells and gene edited derives in which a dual epitope tag was engineered into the amino terminus of the endogenous SMC1A and SMC3 subunits of cohesin. Cohesin complexes were then purified by dual-affinity purification, and Western blot was performed with ubiquitin antibodies (Fig. 5*A*). This experiment demonstrated that endogenous cohesin is ubiquitinated in untransfected HCT116 cells.

Next, we wanted to more specifically define the ubiquitination state of individual cohesin subunits. To do this, we transfected 293T cells with an expression vector for His-tagged ubiquitin, purified ubiquitinated proteins by Ni-NTA affinity chromatography (used to purify His-tagged proteins), and performed Western blot with antibodies to individual cohesin subunits (Fig. 5, *B*–*H*). The effect of transfection of untagged ubiquitin on global protein ubiquitination in 293T cells is shown in Fig. S2. Slower-migrating ubiquitinated forms of cohesin subunits SMC3, SMC1A, STAG2, RAD21, STAG1, PDS5A, and Sororin were readily identified in Ni-NTA affinity purifications from cells expressing His-ubiquitin but not in affinity purifications from cells transfected with vector alone. SMC3, STAG1, PDS5A, and Sororin were primarily mono-ubiquitinated; and SMC1A, STAG2, and RAD21 were both mono- and poly-ubiquitinated. Mono-ubiquitination of proteins is thought to play a wide range of roles in protein regulation, whereas poly-ubiquitination is thought primarily to target proteins for degradation by the proteasome (22). RAD21 was by far the most heavily poly-ubiquitinated protein, an intriguing finding given its known role in regulating the metaphase to anaphase transition *via* its targeted degradation. Together, these results indicated that cohesin subunits are ubiquitinated in cultured human cells.

**Figure 6:**
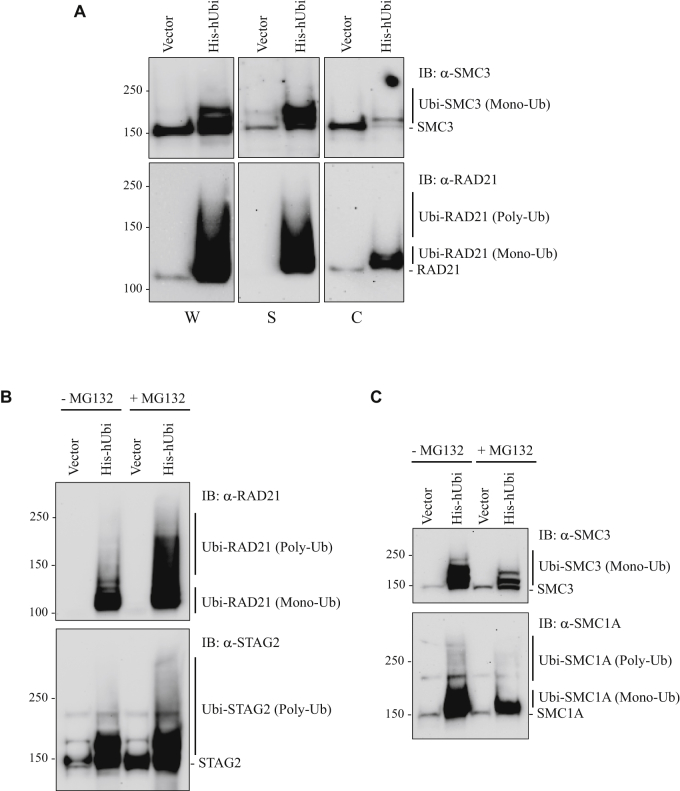
**Ubiquitination of some, but not all, cohesin subunits targets them for degradation by the proteasome.***A*, whole cell lysates (W), Soluble lysates (S), and chromatin lysates (C) were prepared from 293T cells that had been transfected with the His-tagged ubiquitin expression vector (or vector alone) and Ni-NTA affinity purifications were performed, followed by Western blot with SMC3 and RAD21 antibodies. *B*–*C*, 293T cells were transfected with the His-tagged ubiquitin expression vector (or vector alone), then treated with (and without) the MG132 proteasome inhibitor. Ni-NTA affinity purifications were then performed, followed by Western blot with *B*, RAD21 and STAG2 antibodies, and *C*, SMC3 and SMC1A antibodies.

We wondered whether ubiquitinated cohesin subunits were present primarily in the soluble fraction (where the USP13–cohesin complex resides), in the chromatin fraction, or both. To test this, whole cell lysates, soluble lysates, and chromatin lysates were prepared from 293T cells that had been transfected with an expression vector for His-tagged ubiquitin. Ubiquitinated proteins were then purified by Ni-NTA affinity chromatography, and Western blot was performed with SMC3 and RAD21 antibodies as representative examples of mono-ubiquitinated and poly-ubiquitinated cohesin subunits, respectfully (Fig. 6*A*). Slower-migrating, ubiquitinated forms of cohesin subunits SMC3 and RAD21 were much more abundant in the non–chromatin bound, soluble fraction than in the chromatin fraction.

### Ubiquitination targets some, but not all, subunits of cohesin for degradation by the proteasome

We next examined whether ubiquitination of cohesin subunits RAD21, STAG2, SMC1A, and SMC3 targeted them for degradation by the proteasome. To test this, we transfected 293T cells with the His-tagged ubiquitin expression vector, treated the cells with (and without) proteasome inhibitors (MG132), then performed Ni-NTA affinity purification and Western blot with RAD21, STAG2, SMC1A, and SMC3 antibodies. Treating cells with MG132 substantially increased the levels of poly-ubiquitinated RAD21 and STAG2 (Fig. 6*B*), indicating that ubiquitination of RAD21 and STAG2 targeted them for degradation by the proteasome. In contrast, MG132 treatment resulted in the paradoxical decrease in ubiquitinated SMC3 and SMC1A (Fig. 6*C*). Together, these results suggest that ubiquitination of RAD21 and STAG2 leads to their targeted degradation, whereas ubiquitination of SMC3 and SMC1A does not.

### USP13 is required for both deubiquitination and ubiquitination of cohesin subunits

**Figure 7:**
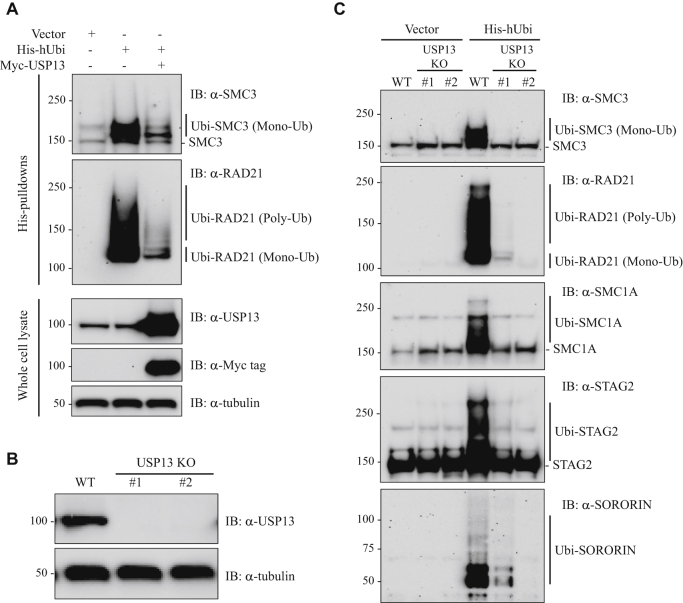
**USP13 is required for ubiquitination of cohesin.***A*, 293T cells were cotransfected with the His-tagged ubiquitin expression vector (His-hUbi) together with an expression vector for myc-USP13. Protein lysates were prepared and Ni-NTA affinity purifications performed followed by Western blot with SMC3 and RAD21 antibodies. *B*, 293T cells were infected with a USP13-KO CRISPR lentivirus. Individual puromycin resistant colonies were obtained by limiting dilution, and loss of USP13 protein in individual homozygous KO clones was demonstrated by Western blot with USP13 antibodies. Parental 293T cell and the two homozygous KO clones used in all subsequent experiments (#1, #2) are shown. *C*, 293T parental cells and USP13 KO clones #1 and #2 were transfected with His-hUbi (or vector alone). Twenty-four hours after transfection, the MG132 proteasome inhibitor was added and the cells were cultured for an additional 4 h. The cells were then lysed in denaturing lysis buffer and sonicated and the ubiquitinated proteins purified by affinity purification on a Ni-NTA column, as described in detail in Experimental Procedures. The ubiquitinated proteins were then separated by SDS-PAGE, and Western blot was performed with antibodies to cohesin subunits SMC3, RAD21, SMC1A, STAG2, and Sororin.

To investigate whether USP13 mediated the deubiquitination of cohesin subunits, we cotransfected 293T cells with expression vectors for His-ubiquitin and Myc-USP13, purified ubiquitinated proteins by Ni-NTA affinity chromatography, and performed Western blot with antibodies to SMC3 and RAD21 (Fig. 7*A*). Ectopic overexpression of USP13 significantly decreased the levels of endogenous ubiquitinated SMC3 and RAD21, indicating that USP13 can function as a deubiquitinase for cohesin subunits.

Because ectopic overexpression of USP13 promoted deubiquitination of cohesin subunits, inactivation of USP13 was conversely expected to cause enhanced accumulation of ubiquitinated cohesin subunits. To test this hypothesis, we used CRISPR to introduce biallelic truncating mutations into the endogenous alleles of USP13 in 293T cells (see Experimental Procedures for details). Complete inactivation of USP13 protein in two independently derived knockout clones was confirmed by Western blot (Fig. 7*B*). Next, 293T parental cells and USP13 KO derivatives were transfected with a His-ubiquitin expression vector, ubiquitinated proteins purified using Ni-NTA beads, and SMC1A, SMC3, STAG2, Sororin, and RAD21 Western blots performed (Fig. 7*C*). As expected, in parental cells there was robust mono- and poly-ubiquitination of these cohesin subunits. Unexpectedly, in both of two independently derived USP13 KO clones, there was a complete loss of ubiquitination in all cohesin subunits tested—SMC1A, SMC3, STAG2, Sororin, and RAD21. The data presented in Figure 7*C* reveal that, despite its known deubiquitinase activity, USP13 is paradoxically required for ubiquitination of cohesin.

### Ubiquitination of cohesin is required for the disassociation of cohesin from chromatin in mitosis

**Figure 8:**
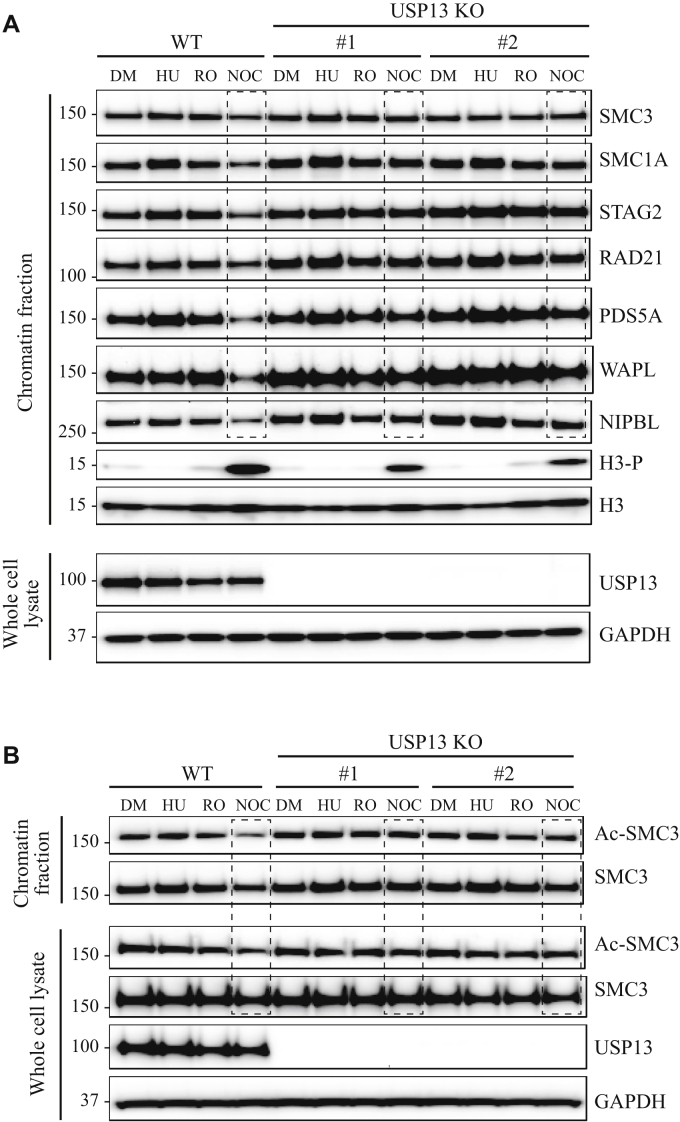
**USP13 is required for release of cohesin from chromatin in mitosis.***A*, 293T parental cells and USP13 KO derivatives #1 and #2 were treated for 20 h with DMSO vehicle alone (DM); hydroxyurea (HU) to arrest cells in the S phase; RO-3306 (RO) to arrest cells in G2; and nocodazole (NOC) to arrest cells in mitosis. Chromatin lysates and whole cell lysates were prepared, and Western blot was performed with the antibodies to cohesin subunits SMC3, SMC1A, STAG2, RAD21, PDS5A, WAPL, NIPBL, and Sororin, as well as the control antibodies indicated. *B*, same as for (*A*) except Western blots were performed with antibodies to Ac-SMC3 and SMC3, as well as the control antibodies indicated. Quantification of Western blot bands is shown in Fig. S3.

In human cells, cohesin is loaded onto chromatin in telophase and G1 and removed in mitosis. We hypothesized that ubiquitination might regulate this cell cycle–dependent interaction of cohesin with chromatin. To test this, parental 293T cells and isogenic USP13 KO derivatives were arrested in the S, G2, or M phase of the cell cycle by treating them with hydroxyurea, RO-3306, or nocodazole, respectively, for 20 h. Whole cell lysates and chromatin lysates were prepared, and Western blot was performed with antibodies to cohesin subunits. As shown in Figure 8*A* and quantified in Fig. S3*A*, there was no effect of USP13 on the interaction of cohesin subunits with chromatin in asynchronous cells or in cells arrested in S phase or G2. However, there was an effect of USP13 inactivation on the interaction of cohesin with chromatin in mitotic cells. Cohesin was released from mitotic chromatin in USP13-proficient cells, whereas this release did not occur in USP13-deficient cells.

The SMC3 subunit of cohesin is acetylated in the S phase, which is important for the establishment of sister chromatid cohesion. Acetylated-SMC3 (ac-SMC3) dissociates from chromosome during mitosis and then is deacetylated by HDAC8 (23). We next sought to determine whether ubiquitination of cohesin effects the dissociation of ac-SMC3 from chromosomes in mitosis. As shown in Figure 8*B* and quantified in Fig. S3*C*, the level of ac-SMC3 was decreased in chromatin fractions prepared from wildtype cells treated with nocodazole as expected, indicating that ac-SMC3 dissociates from chromosomes. Inactivation of USP13 resulted in the retention of ac-SMC3 on chromatin in mitosis (Fig. 8*B*). These data indicate that ubiquitination of cohesin is required for the dissociation of ac-SMC3 from chromosomes during mitosis.

## Discussion

In this study we have identified and validated USP13 as a functionally significant new cohesin-interacting protein in human cultured cells. We further show that USP13 is required for the ubiquitination of cohesin complexes and that this is required for the release of cohesin from chromatin during mitosis.

In addition to identifying USP13 as a new cohesin-interacting protein, we have also provided a comprehensive analysis of the ubiquitination state of endogenous cohesin subunits in cultured human cells. This analysis revealed that most subunits of cohesin harbor prominent, easily detected mono-ubiquitination, including SMC1A, SMC3, STAG1, STAG2, PDS5A, and Sororin. Several subunits also showed evidence of poly-ubiquitination, most prominently, RAD21. Of note, poly-ubiquitination is generally thought to target proteins for degradation by the proteasome, whereas mono-ubiquitination of proteins is thought to target them for a wide variety of other regulated cellular outcomes (22). Further experiments are needed to define the specific regulatory effects of mono-ubiquitination on individual subunits of cohesin.

It was unexpected that deletion of USP13 resulted in the complete loss of cohesin ubiquitination, rather than the enhanced cohesin ubiquitination that would be expected after inactivation of a deubiquitinase. Although we do not at this point understand the mechanism for this, we speculate that there is a complex interplay between the enzymes that result in the ubiquitination and deubiquitination of cohesin such that inactivation of the deubiquitinase (USP13) results in the effective inactivation of the putative cohesin ubiquitin ligase as well. However, testing this hypothesis will require the identification of the cohesin ubiquitin ligase, which has not yet been accomplished.

It was also surprising that, despite being essential for cohesin ubiquitination, USP13 is dispensable for viability and proliferation in multiple human cell lines. This finding, together with the sister chromatid cohesion assays presented herein, suggests that USP13-mediated ubiquitination of cohesin subunits is not required for their canonical activity in controlling cell cycle progression (or in their more recently defined essential function as engines for chromatin loop extrusion (24, 25)). Therefore, we speculate that USP13 controls the ability of cohesin to regulate the more subtle aspects of cellular differentiation and organismal development. Such a hypothesis would be consistent with the finding that no known USP13 ortholog exists in unicellular organisms such as *S. cerevisiae*. To test this hypothesis, studies of the intersections of USP13 and cohesin in cellular differentiation and organismal development are currently underway.

We are also intrigued by the notion that cohesin may be classified as a multiprotein complex with not only loop extrusion catalytic activity but also inherent USP13-dependent deubiquitinase activity as well. Our preliminary studies indicate that cohesin complexes purified from the soluble nuclear fraction *do* have inherent deubiquitinase activity (unpublished data). In this study we have focused on ubiquitination of the cohesin complex itself as a USP13-regulated target; however, it is likely that USP13-bound cohesin regulates the ubiquitination of other protein targets as well. Further experiments to address a novel potential role as cohesin as a multiprotein, cell-cycle regulated deubiquitinase seem warranted.

It is worth noting that a previous study implicated a different deubiquitinase, USP37, as interacting with and regulating the ubiquitination of cohesin (12). Our exhaustive mass spectrometry analysis of endogenous cohesin complexes, described in ref. (13), failed to confirm the existence of this reported interaction. This discrepancy may be due to cell type–specific or technical differences, since the reported interaction between USP37 and cohesin required ectopic expression of at least one component of the putative complex.

In conclusion, here we demonstrate the existence of a robust, functionally and biochemically significant physical interaction between endogenous cohesin and endogenous USP13 in the soluble nuclear fraction of human cells. Further studies are required to reveal the details of the biomolecular interactions within the complex and its importance in the various critical functions of cohesin and ubiquitination in cell biology and human disease.

## Experimental procedures

### Cell lines

293T, HCT116, HeLa, and RPE-hTERT cells were obtained from ATCC. Cells were maintained in Dulbecco's modified Eagle's medium supplemented with 10% fetal bovine serum and 1% Pen/Strep at 37 °C in 5% CO_2_.

### Plasmids

pCI-His-hUbi was a gift from Astar Winoto (Addgene #31815; ref. (26)). pRK5-Myc-USP13 was made by replacing the FLAG tag in pRK5-FLAG-USP13 (a gift from Yihong Ye, NIH; ref. (19)) with a Myc tag. Mutant forms of USP13 (ΔZnF, ΔUBA, ΔZnFΔUBA, and USP) were created by cloning the mutant inserts from pME-FLAG-USP13 (a gift from Masayuki Komada; ref. (27)) into the plasmid pRK5-Myc-USP13. All mutations were confirmed by DNA sequencing.

### Antibodies

Primary antibodies for immunoblotting were FLAG (M2) and tubulin (DM1α) from Sigma-Aldrich; Sororin (ab192237) from Abcam; STAG2 (sc-81852), NIPBL (sc-374625), and ubiquitin P4D1 (sc-8017) from Santa Cruz Biotechnology; USP13 (12577), GAPDH (2118), Histone H3 (14269), and Phospho-H3 (53348) from Cell Signaling Technologies; PDS5A (A300-088), RAD21 (A300-080), SMC1A (A300-055), SMC3 (A300-060), and STAG1 (A302-579) from Bethyl Laboratories; and ubiquitin (1859660) from Thermo Fisher.

### Protein preparation

Nuclear extracts for dual-affinity purification followed by GeLC-MS/MS were prepared using a modification of Dignam's nondetergent lysis method (28, 29). Whole cell protein lysates for immunoprecipitation and Western blotting were prepared in either NP40 lysis buffer (50 mM Tris-HCl pH 7.5, 150 mM NaCl, 1% NP40) or RIPA buffer (50 mM Tris-HCl pH 7.5, 150 mM NaCl, 1% NP40, 0.5% sodium deoxycholate, 0.1% SDS). Chromatin-associated proteins and total soluble proteins were prepared as described (30). Protein concentrations were determined using the bicinchoninic assay (Pierce).

### Immunoprecipitation

Whole cell lysates (500 μg) were incubated with 1 μg of SMC3 antibody, Myc antibody, or IgG control antibody in TBS buffer (50 mM Tris-HCl pH 7.5, 150 mM NaCl) containing 0.1% NP-40 at 4 °C overnight with rotation. Protein A/G agarose beads (Santa Cruz Biotechnology) were then added to the lysates and further incubated for 1 h at 4 °C with rotation. Beads were then washed three times with TBS containing 0.1% NP-40. Proteins were eluted into 2X LDS sample buffer at room temperature for 20 min. Samples containing 50 mM DTT were boiled for 5 min and separated by SDS-PAGE.

### Western blot

Proteins were separated by SDS-PAGE on 3% to 8% Tris-acetate gels or 4% to 12% Bis-Tris gels (Thermo Fisher). Proteins were transferred to polyvinylidene difluoride membranes and then probed with a 1:1000 dilution of primary antibodies overnight at 4 °C with rotation followed by incubation with horseradish peroxidase–conjugated secondary antibodies (Cell Signaling) for 1 h at room temperature. The membranes were developed with Super Signal West Pico PLUS chemiluminescent substrate (Pierce) and imaged using a myECL imager (Thermo Fisher).

### AAV-mediated gene editing

AAV-based gene editing was used to modify an endogenous allele of USP13. The biological and technical principles underlying AAV-based gene editing are described in detail in refs. (31, 32) and (33).

Gene editing vectors were designed as shown in Figure 2*A*. Homology arms were synthesized by Genscript and cloned sequentially into pAAV-SEPT, an AAV-based gene editing acceptor vector we previously reported in which polylinkers for the cloning of LHAs and RHAs flank a promoterless splice-acceptor-IRES-neo^R^ gene (32). Next, transient stocks of AAV-2 virions were created by cotransfection of 293T cells with gene editing vectors together with pAAV-RC (Stratagene) and pHELPER (Stratagene) using X-tremeGENE 9 (Roche). Two days after transfection, the medium was aspirated and cell monolayers were scraped into 1 ml PBS and subjected to four cycles of freeze/thaw. The lysate was then clarified by centrifugation at 12,000 rpm for 10 min in a benchtop microfuge to remove cell debris, and the virus-containing supernatant was aliquoted and stored at −80 °C.

The virus was then used to infect HCT116 cells in a T25 tissue culture flask, and 24 h later, the cells were passaged at limiting dilution into 96-well plates in the presence of 1.0 mg/ml G418. After 14 days, individual G418-resistant clones were expanded and genomic DNA was prepared in a 96-well format using the QIAamp 96 DNA Blood kit (Qiagen). The clones were tested for homologous integration of the targeting vector using a primer pair specific for the targeted allele. Homologous integration of the dual epitope tag was then confirmed by DNA sequencing. The gene-edited clones were then infected with cre-expressing adenovirus overnight and plated at limiting dilution into 96-well plates. Single colonies were expanded and tested for G418 sensitivity to identify clones in which the gene-edited allele had been restored to its otherwise natural configuration. Homology arm and primer sequences are available from the authors upon request.

### Dual-affinity purification

Nuclear extracts were prepared from ∼10^9^ cells (∼60 confluent 15 cm dishes) for parental HCT116 cells and USP13 epitope-tagged derivatives. Next, 60 mg nuclear extract was incubated with FLAG M2 beads (Sigma) rotating at 4° for 1 h. The beads were then transferred to Poly-Prep chromatography columns (Bio-Rad) and washed with 30x bed volume Tris-buffered saline (TBS) containing 150 mM NaCl. The proteins were then eluted with 5x bed volume 100 ng/μl 1x FLAG peptide (Sigma). FLAG eluents were then applied to Streptavidin Plus UltraLink resin (Pierce) rotating at 4° for 1 h. The beads were then transferred to Poly-Prep chromatography columns and washed with 30x bed volume TBS containing 150 mM NaCl. The proteins were then eluted with 2x bed volume 1 mM D-biotin and then concentrated by TCA precipitation, dissolved in LDS Sample Buffer (Thermo Fisher), and subjected to GeLC-MS/MS.

### GeLC-MS/MS

Dual-affinity purified proteins were separated by SDS-PAGE. The gels were fixed in 50% methanol/7% acetic acid for 1 h at RT, washed with water, stained with Coomassie Brilliant Blue (Thermo Fisher) for 2 h, and destained with water overnight. Each lane was then excised into seven pieces. Gel slices were washed in 50% acetonitrile and rehydrated with a 50 mM ammonia bicarbonate/trypsin solution and digested at 37 °C overnight. Peptides were then extracted with a series of elutions, dried in a speed vac, and solubilized in 0.1% formic acid in water for analysis by tandem mass spectrometry.

LC-MS/MS was performed on a LTQ Orbitrap Elite (Thermo Fisher) equipped with a Waters (Milford, MA, USA) NanoAcquity HPLC pump as described (13). Primary mass spectrometry data have been submitted to the MASSIVE database (MSV000086356) and PRIDE repository (PXD022135).

### Double thymidine block cell synchronization

293T cells at 50% confluence were treated with 2 mM thymidine (Sigma) for 20 h to arrest cells in S phase. The cells were then released by washing in Hank’s Buffered Saline Solution followed by an 8-h incubation in regular media. The cells were then treated again with 2 mM thymidine for 16 h to generate a synchronized population of cells arrested in the S phase. They were then washed in Hank’s Buffered Saline Solution, incubated in regular media to release them from the cell cycle arrest, and harvested at different time points for flow cytometry and preparation of whole cell lysates.

### Drug treatments for cell cycle arrest

Hydroxyurea (Sigma) was used at a final concentration of 0.5 mM. RO-3306 (Sigma) was used at a final concentration of 10 μM. Nocodazole (Sigma) was used at a final concentration of 100 ng/ml.

### Flow cytometry

Cells were fixed in 70% ethanol and stained in PBS containing 0.1% Triton X-100, 50 μg/ml RNase, and 50 μg/ml propidium iodide. The DNA content was measured on a FAC Sort flow cytometer (Becton Dickinson), and data were analyzed using ModFit software (Verity Software House).

### Ubiquitination assays

293T cells (2 × 10^6^) were seeded in 10-cm cell culture dishes and transfected with 2 μg of plasmid encoding His-ubiquitin or empty vector using X-tremeGENE 9 (Roche Applied Science). At 24 h after transfection, the proteasome inhibitor MG132 was added to a final concentration of 25 μM for an additional 4 h. Cells were then washed twice with cold PBS and lysed in 1 ml of denaturing lysis buffer (6 M quanidine-HCl, 10 mM Tris-HCl pH8.0, 100 mM Na_2_HPO_4_, 10 mM imidazole, and 10 mM β-mercaptoethanol). The lysates were then sonicated twice for 10 s each to shear DNA. After sonication, 50 μl of Ni-NTA agarose beads (QIAGEN, Cat No. 30210) were added to the cell lysate and incubated at 4 °C for 2 h while rotating. The beads were then washed twice with 1 ml of denaturing lysis buffer containing 0.1% Triton X-100, twice in 1 ml of wash buffer A (8 M urea, 100 mM NaH_2_PO_4_, 10 mM Tris-HCI, pH 6.8, 10 mM imidazole, 0.1% Triton X-100, and 10 mM β-ME), and twice in wash buffer B (100 mM Na_2_HPO_4_, 10 mM Tris-HCI, pH 6.8, 30 mM imidazole, 0.1% Triton X-100, 10 mM β-mercaptoethanol). The beads were then incubated with 35 μl of elution buffer A (50 mM NaH_2_PO_4_, 250 mM imidazole, pH 6.8, 2X LDS sample buffer, and 5% β-ME) for 20 min at room temperature and then centrifuged in a microfuge at 7000 rpm for 1 min at room temperature. The supernatant was then transferred into a new microfuge tube, DTT was added to a final concentration of 50 mM, and samples were boiled before being analyzed by Western blot.

### CRISPR gene editing

To introduce biallelic truncating mutations in the endogenous alleles of USP13 using CRISPR, a guide RNA targeting the first coding exon of USP13 was designed using the Feng Zhang lab CRISPR guide RNA design tool (crispr.mit.edu) and cloned into lentiCRISPR v2 (a gift from Feng Zhang, Addgene #52961, ref. (34)). Lentivirus was packaged using pMD2.G (a gift from Didier Trono, Addgene #12259) and psPAX2 (a gift from Didier Trono, Addgene #12260) and used to infect 293T, HCT116, and HeLa cells. Individual puromycin-resistant colonies were obtained by limiting dilution, and loss of USP13 protein was validated by Western blotting. The presence of homozygous truncating mutations was confirmed by DNA sequencing.

### Sister chromatid cohesion assays

Cells were arrested in mitosis by culturing in the presence of 500 nM nocodazole. Chromosome spreads were then performed by subjecting the cells to hypotonic treatment, followed by fixation with Carnoy’s fixative, spreading on glass slides, and Giemsa staining. Micrographs were obtained using a DeltaVision microscope (Applied Precision) fitted with a U-PlanApo 100X Oil Objective, 1.40 NA, 0.12 mm WD (Olympus) and a CoolSnap HQ2 camera (Photometrics).

## Data availability

Primary mass spectrometry data generated and reported in this article are available in the MASSIVE database (MSV000086356) and PRIDE repository (PXD022135). All other data are contained within the article and in the supporting information.

## Conflict of interest

The authors declare that they have no conflicts of interest with the contents of this article.
