# Supplementary material for: USP13 interacts with cohesin and regulates its ubiquitination in human cells
Source: J Biol Chem. 2020 Dec 20;296:100194. doi: 10.1074/jbc.RA120.015762 (PMC7948425; doi:10.1074/jbc.RA120.015762)
Supplement: Figures S1–S3 [file mmc1.pdf]

Supporting Information

USP13 interacts with cohesin and regulates its ubiquitination in human cells

Xiaoyuan He, Jung-Sik Kim, Laura Diaz-Martinez, Cecil Han, William S. Lane, Bogdan Budnik, and Todd Waldman

Supplementary Figures

Supplementary Figure 1
Supplementary Figure 2
Supplementary Figure 3

Supplementary Fig. 1

A

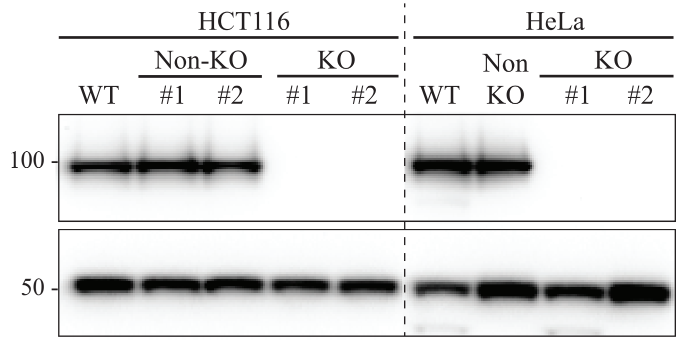

B

Cells		Cohesion assay			Mitotic Index		
		Cells with normal cohesion	Cells with cohesion defects	% Cohesion defects	Mitosis	Interphase	Mitotic Index (%)
HCT116	HCT116 WT	316	4	1.25	141	381	27.0
	non-KO #1	400	3	0.74	152	426	26.3
	non-KO #2	313	2	0.63	113	321	26.0
	USP13 KO #1	441	2	0.45	113	323	25.9
	USP13 KO #2	309	0	0.00	59	324	15.4
HeLa	HeLaWT	335	3	0.89	58	333	14.8
	non-KO	361	8	2.17	54	338	13.8
	USP13 KO #1	362	7	1.90	79	403	16.4
	USP13 KO #2	400	9	2.20	91	537	14.5

Supplementary Figure 1. USP13 is dispensable for sister chromatid cohesion in human cells. *A,* HCT116 and HeLa cells were infected with a USP13-KO CRISPR lentivirus. Individual puromycin resistant colonies were obtained by limiting dilution, and loss of USP13 protein in individual homozygous KO clones was demonstrated by Western blot with USP13 antibodies. Parental HCT116 and HeLa cells as well as two independently-derived homozygous KO clones used in subsequent experiments are shown. *B*, Sister chromatid cohesion assays were performed on the cell lines described in (*A*), as described in detail in Experimental Procedures.

Supplementary Fig. 2

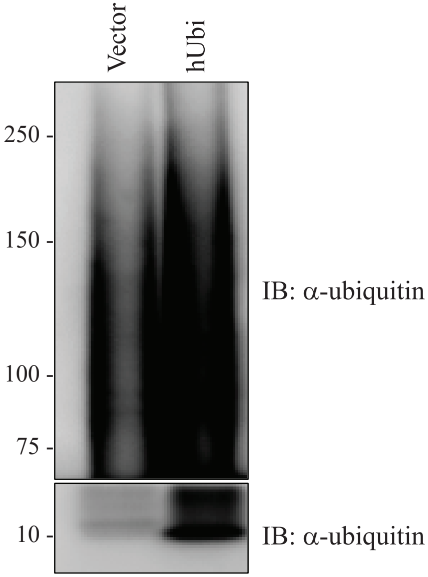

Supplementary Figure 2. Effect of transfection of untagged ubiquitin on protein ubiquitination in 293T cells. 293T cells were transfected with an untagged ubiquitin expression vector or with vector alone. Whole cell lysates were prepared 24 h after transfection, and Western blot performed with ubiquitin antibodies (Santa Cruz).

Supplementary Fig. 3

A

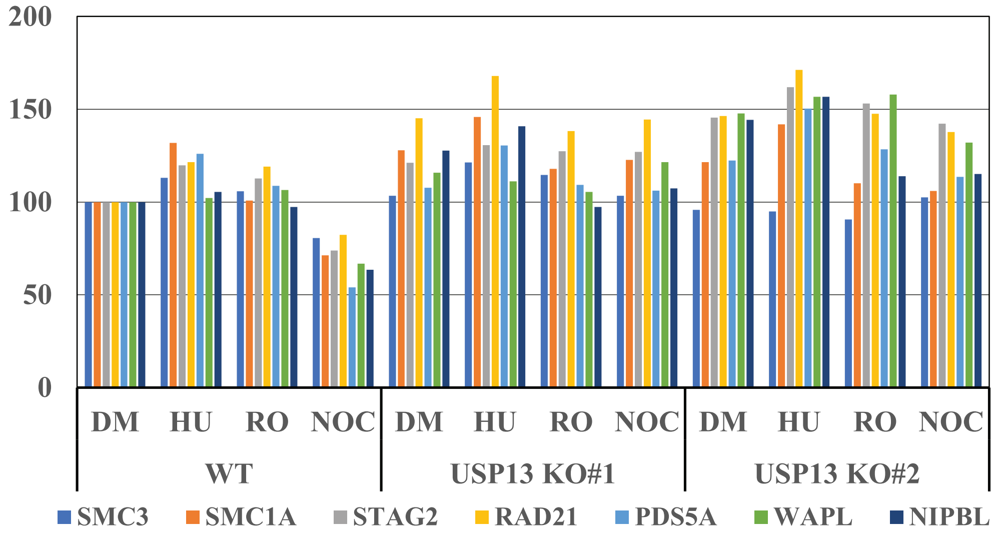

B

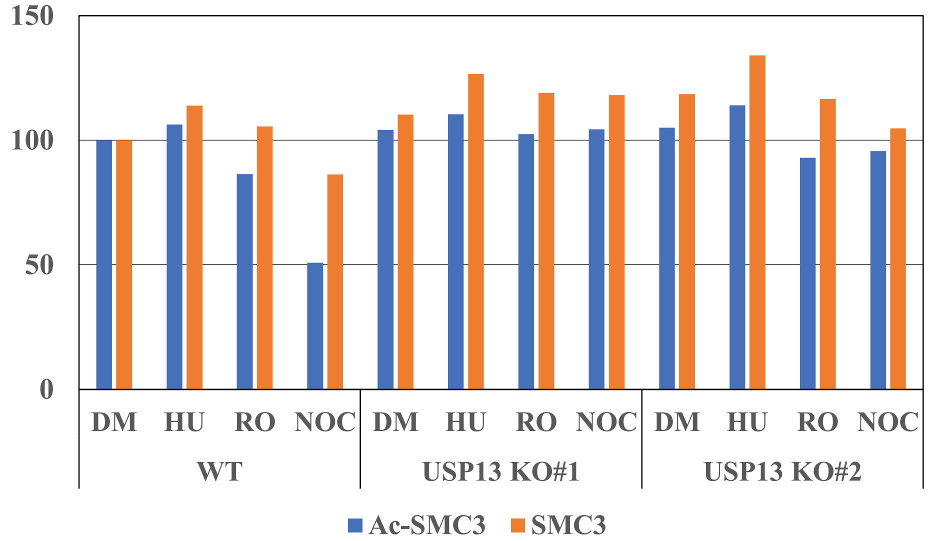

Supplementary Figure 3. USP13 is required for release of cohesin from chromatin in mitosis. *A*, Quantification of bands in Fig. 8A using ImageJ. The amount of chromatin-bound cohesin subunits SMC3, SMC1A, STAG2, RAD21, PDS5A, WAPL, and NIPBL is reduced during mitosis in cells with wild-type USP13, but not reduced in mitosis in two independently-derived isogenic USP13 KO clones. *B*, Same as *A* above but for Ac-SMC3.
